# 

**Published:** 1906-08-11

**Authors:** 


No. 1,038. Vol. XL. [Registered as a Newspaper.] SATURDAY, AUGUST 11TH, 1906. [Subscription Terms see p. 338] PRICE THREEPENCE

